# Effective, polyvalent, affordable antivenom needed to treat snakebite in Nepal

**DOI:** 10.2471/BLT.17.195453

**Published:** 2017-08-01

**Authors:** Bhola R Shrestha, Deb P Pandey, Krishna P Acharya, Chhabilal Thapa-Magar, Fahim Mohamed, Geoffrey K Isbister

**Affiliations:** a Curative Service Division, Ministry of Health and Population, Kathmandu, Nepal.; b South Asian Clinical Toxicology Research Collaboration, Faculty of Medicine, University of Peradeniya, Peradeniya, Sri Lanka.; c Bheri Zonal Hospital, Nepalgunj, Nepal.; d Kaligandaki Health Foundation, Kawasoti, Nepal.; e Clinical Toxicology Research Group, University of Newcastle, Newcastle, Australia.

Nepal has one of the highest snakebite fatality rates in south Asia. A study in the country in 2001 indicated an annual incidence, in the study area, of 162 snakebite-related deaths per 100 000 population.^1^ Between 1987 and 2013, in other studies in Nepal, snakebite fatality rates ranging between 3% (2 deaths out of 71 envenomed people) and 58% (28 deaths out of 48 envenomed people) were reported.^2^ An epidemiological survey of 15 hospitals indicated that, nationwide, approximately 20 000 snakebite cases and about 1000 snakebite-related deaths were occurring each year.^3^ Many of the reported rates underestimate the true burden posed by venomous snakes in Nepal because data recording in Nepalese hospitals is generally poor^4^ and many Nepalese depend on traditional healers and or do not seek hospital treatment because they consider themselves to be too poor to pay for treatment, or think there will be no benefit.^2^^,^^5^ If the numbers of out-of-hospital deaths caused by snakebite are to be reduced in Nepal, there needs to be improvements in the public awareness of the benefits of snakebite treatment in hospital and in the pre-hospital care of snakebite – as seen, for example, in Sri Lanka.^6^ The corresponding in-hospital mortality will only be reduced by increasing the availability of safe and effective antivenoms and improving critical care for people bitten by snakes.

Even though at least 70 snake species are known to exist in Nepal, most of the serious envenoming and deaths from snakebite are caused by just seven species: the kraits *Bungarus caeruleus*, *B. walli*, *B. lividus* and *B. niger*, the cobras *Naja naja* and *N. kaouthia* and the viper *Daboia russelii.*^7^^,^^8^ Although hospital data indicate that just 3% of snakebites (10 out of 349 recorded snakebites) in Nepal are caused by pit vipers,^7^ many people bitten are never admitted to hospitals because the resultant envenoming is usually mild and or the bitten individuals prefer to seek care from traditional healers. However, some pit viper bites can cause severe coagulopathy.^9^ In Nepal, antivenom for treating pit viper bites is currently unavailable, but two antivenoms, raised against pit vipers from other countries, were used successfully, in Australia, to treat a pit viper bite that occurred in Nepal.^9^

## Antivenom shortages

Antivenom is generally in short supply in most parts of Africa and many parts of Asia.^10^ As the safe production of efficacious antivenoms becomes generally more expensive and less profitable for the manufacturers and are either discontinuing the production of some antivenoms or only producing them in very limited amounts.

In 2012, an Indian court order decreased the export of antivenom manufactured in India to Nepal until sufficient quantity of antivenom was available to meet the requirement needs of all Indian citizens. This order led to a reduction in the availability of antivenom in Nepal.^11^ To address the shortfall, the Nepalese government aims to begin its own production of antivenom. However, the achievement of this goal is hampered by the lack of adequate relevant information on snakebite epidemiology, clinical studies and assessments of the economic costs of antivenom production. The treatment of snake envenoming in Nepal is mostly reliant on a single polyvalent antivenom produced, in Indian horses, against the venoms of Indian *B. caeruleus*, *D. russelii*, *Echis carinatus* and *N. naja*.

Until Nepal is able to produce antivenom of high quality, antivenoms will need to be imported from other Asian countries or elsewhere. As a temporary measure, i.e. until local production is ready in terms of capacity and funding, antivenoms produced by competent non-profit institutions, e.g. in Brazil or Costa Rica,^12^ could be imported.

Even antivenoms produced in neighbouring India may be less efficacious against some snake species in Nepal than against the same species in India because of geographical intraspecific differences in venom composition.^13^ Such differences may explain reports of the low efficacy or failure of Indian polyvalent antivenom in the neutralization of Sri Lankan snake venoms.^13^^,^^14^ The efficacy of the Indian antivenom in neutralizing the venoms from all of the species of venomous snake in Nepal, not just those from *B. caeruleus*, *D. russelii*, *E. carinatus* and *N. naja* – needs to be assessed in both in vitro studies and comprehensive clinical trials. The results of such assessments may help in the Nepalese government’s attempts to design a new and improved polyvalent antivenom specifically for use in Nepal.

## Antivenom production in Nepal

To develop a new and cost–effective antivenom, the Nepalese government will need to develop sufficient infrastructure, including snake farms for the provision of venom, horses or other appropriate animals that can be injected with the venoms and laboratory facilities for the preparation of safe antivenoms.

In Nepal, as a single vial of imported antivenom costs the equivalent of US$ 19 (United States dollars) and about 40 000 such vials are used annually,^15^ there is a considerable potential market for a new antivenom. The production of antivenom tailored specifically to the needs of Nepal would be expected to reduce snakebite-related morbidity and mortality in the country. However, the production of such antivenom is unlikely to be profitable and, without the support of humanitarian organizations, it is unlikely to be sustainable. Therefore, increased awareness, greater political will, adequate financial support and vested interest from the public health sector are needed. In addition, there will need to be collaboration with national and international experts in the field and quality research to determine the appropriate use, effectiveness and safety of any new antivenom in Nepal.

Stakeholders will need to acquire certain information before developing and producing a new antivenom in Nepal. First, stakeholders should do a formal assessment of the impact of snake envenoming in the country, overall improvement of the treatment of snakebite and the relevant national guidelines. They should conduct epidemiological studies of snake envenoming across the country, both to identify the snake species of greatest medical importance and current reaction rates to any Indian antivenom being used. Also, improved data recording of snakebites in hospitals is needed. Second, stakeholders should make a comparison of the cost–effectiveness between the Indian antivenoms in current use with that of any new Nepalese antivenom, which is likely to be more expensive but may cause fewer adverse reactions. Stakeholders should consider that launching the production of a new antivenom in Nepal may be problematic and pose a substantial financial burden for the country.
